# Evaluation of a Modified Objective Structured Assessment of Technical Skills Tool for the Assessment of Pediatric Laceration Repair Performance

**DOI:** 10.7759/cureus.4056

**Published:** 2019-02-12

**Authors:** Neil G Uspal, Anita A Thomas, Rebekah Burns, Maya Jones, Isabel T Gross, Ryan D Kearney, Rachel E Whitney, Julie E Uspal, Nancy Gove, Jennifer Reid

**Affiliations:** 1 Pediatrics, Seattle Children's Hospital - University of Washington School of Medicine, Seattle, USA; 2 Pediatrics, Seattle Children's Hospital- University of Washington School of Medicine, Seattle, USA; 3 Pediatrics, Yale University School of Medicine, New Haven, USA; 4 Emergency Medicine, University of Pennsylvania, Philadelphia, USA; 5 Miscellaneous, Seattle Children's Hospital - University of Washington School of Medicine, Seattle, USA; 6 Emergency Medicine, Seattle Children's Hospital - University of Washington School of Medicine, Seattle, USA

**Keywords:** laceration repair, assessment, pediatric emergency medicine, simulation, task trainers

## Abstract

Introduction

The Accreditation Council for Graduate Medical Education (ACGME) has developed milestones including procedural skills under the core competency of patient care. Progress in training is expected to be monitored by residency programs. To our knowledge, there exists no tool to evaluate pediatric resident laceration repair performance.

Methods

The Objective Structured Assessment of Technical Skills was adapted to evaluate resident laceration repair performance using two components: a global rating scale (GRS) and a checklist. Pediatric and family medicine residents at a tertiary care children's hospital were filmed performing a simulated laceration repair. Videos were evaluated by at least five physicians trained in laceration repair. Concordance correlation coefficients (CCC) were calculated for the GRS and checklist scores. Scores for each resident were compared across levels of training and procedural experience. Spearman's rank order correlations were calculated to compare the checklist and GRS.

Results

Thirty residents were filmed performing laceration repair procedures. The CCC showed fair concordance across reviewers for the checklist (0.55, 95% CI: 0.38–0.69) and the GRS (0.53, 95% CI: 0.36–0.67). There was no significant difference in scores by self-reported experience or training level. There was correlation between the median GRS and checklist scores (Spearman ρ = 0.730, p < .001).

Conclusions

A novel tool to evaluate resident laceration repair performance in a pediatric emergency department showed fair agreement across reviewers. The study tool is not precise enough for summative evaluation; however, it can be used to distinguish between trainees who have and have not attained competence in laceration repair for formative feedback.

## Introduction

The Accreditation Council for Graduate Medical Education (ACGME) has developed milestones for trainees, which include procedural or technical skills under the core competency of patient care [1]. The ACGME Pediatric Residency Review Committee states laceration repair is a procedure pediatric residents should receive training on, and progress in competency should be monitored by pediatric residency programs [2]. Clinical competence has previously been established by procedural experience in the form of procedure logs. However, it has been demonstrated that resident clinical experience is not an accurate proxy for skill [3]. Also, to our knowledge there are no validated tools for evaluation of laceration repair skills in non-surgical residents.

The Objective Structured Assessment of Technical Skills (OSATS) is a tool developed for evaluation of surgical technical skills, including suturing. It consists of checklist and global rating scale (GRS) components in the evaluation of surgical trainees [4-8]. Similar tools have been developed for the evaluation of emergency medicine residents' management of pediatric airways and pediatric residents' performance of lumbar punctures [9-11]. While suturing technique is a central component of both surgery and pediatric emergency department (PED) laceration repair, there are significant differences between the two areas of practice. PED physicians suture less frequently in less controlled environments than surgeons. It is therefore unclear if tools used to evaluate surgical technique are applicable to PED trainees.

To address the above-described need, we developed and evaluated the validity of a modified OSATS tool for the evaluation of residents' laceration repair skills in the PED. Validity, as described by Messick, refers to evidence that an assessment is an accurate and complete representation of a specific construct [12]. A tool is not “valid” or “invalid”; instead, evidence is obtained that either supports or refutes the concept of validity [13]. Sources of validity evidence are content, response process, internal structure, relationship to other variables, and consequences [13]. In this paper, we provide content evidence for our tool by discussing its development. We examine the internal structure of the tool by examining the reproducibility of assessment scores across raters, and compare the performances of components and individual items in the tool. We assess the relationship of tool scores to variables presumed to have a relationship to procedural competence, level of training and procedural experience. Finally, we use this data to establish the appropriate level of consequence for the results obtained by using this assessment tool.

## Materials and methods

This was a study to prospectively evaluate the validity evidence of a novel tool for the evaluation of pediatric resident laceration repair performance. Procedures were performed on suturing task trainers and evaluated via blinded video review. Procedures were recorded at a primary single center, while video reviewers were from multiple centers. The study was reviewed and approved by the Institutional Review Board at the primary study institution, while the study was exempted from review by the Institutional Review Boards at the other study institutions.

Tool development

**Figure 1:**
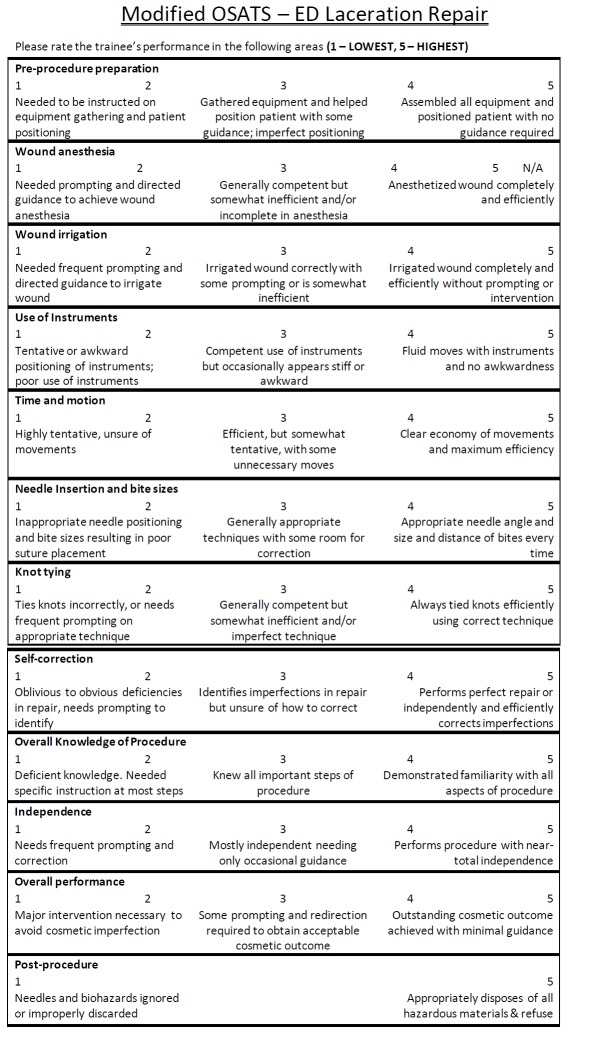
Global rating scale.

**Figure 2:**
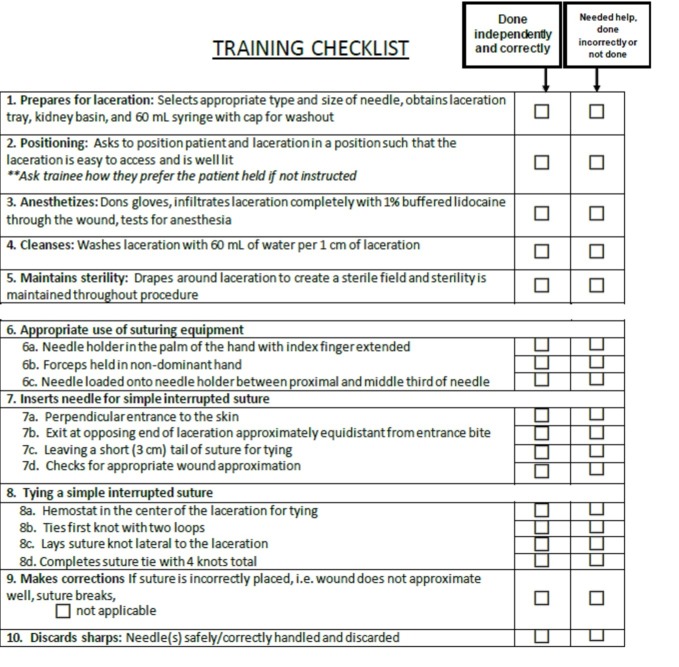
Modified checklist tool.

The modified OSATS tool developed for this study was adapted from OSATS tool used to measure technical skills of surgical residents [5]. Like the OSATS, the modified OSATS tool contains both checklist and GRS components (Figures 1, 2), the content of which was adapted for PED practitioners from published resources on laceration repair [14-16]. Changes to the checklist included items on completion of pre- and post-suturing tasks, as well as inclusion of laceration repair specific tasks and removal of surgery specific tasks. Changes to the GRS also included evaluation of pre- and post-suturing procedures, specific evaluation of needle insertion and bite size and knot tying techniques, and omission of knowledge of tools and use of assistants. The checklist component allows for measurement of knowledge and completion of tasks associated with the laceration repair procedure, while the GRS tool is designed to capture proceduralists' proficiency and efficiency in performing procedural tasks. Both tools were designed to evaluate the entire construct of laceration repair, including pre-procedure preparation, wound irrigation and anesthesia, suturing, and post-procedure clean-up. While at the test site all proceduralists were scored on the proper application of both topical and injected anesthetics, the authors recognize that some centers have a standard of wound care where topical anesthetics alone are employed. Thus, a choice of N/A was included under Wound Anesthesia to support the generalization of the tool.

The modified tool was primarily written by NU, and was reviewed by AT and JR for content validity. The GRS tool was piloted by several pediatric emergency medicine (PEM) physicians evaluating laceration repairs performed by resident physicians during clinical encounters, while the checklist tool was piloted as an evaluative tool for resident just-in-time training by several PEM physicians. Feedback was solicited from PEM physicians who had used the tools, and modifications were made to the tools based on this feedback.

Study recruitment, simulation, and video recording

Study subjects were recruited from the pediatric residents at the study institution and family medicine residents rotating through the study institution's pediatric emergency department. Participants were enrolled in the study between January and November of 2016. Participants were volunteers who were recruited through in-person appeals at resident conferences and targeted e-mails to residents rotating in the pediatric emergency medicine department. Data was collected on residents' area of specialty, training year, and self-estimated number of lacerations previously repaired in an acute care setting. Subjects received a $5 Starbucks gift card as compensation for participating in the study.

**Figure 3:**
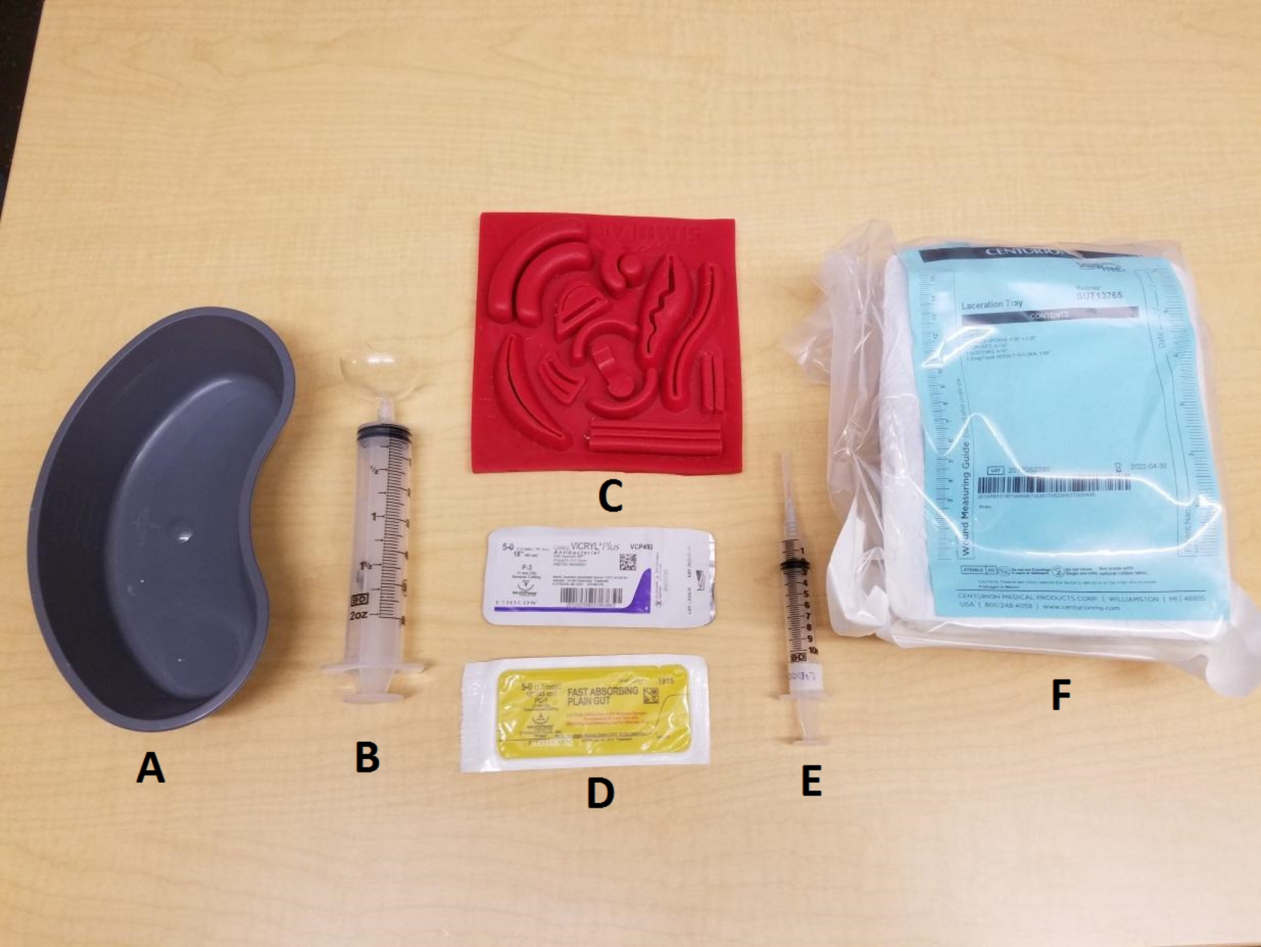
Standardized equipment set-up for simulated laceration repair procedure. A - kidney basin B - 60cc syringe and splash guard C - suture pad D - suture material E - 10cc "lidocaine" syringe F - suture kit

**Figure 4:**
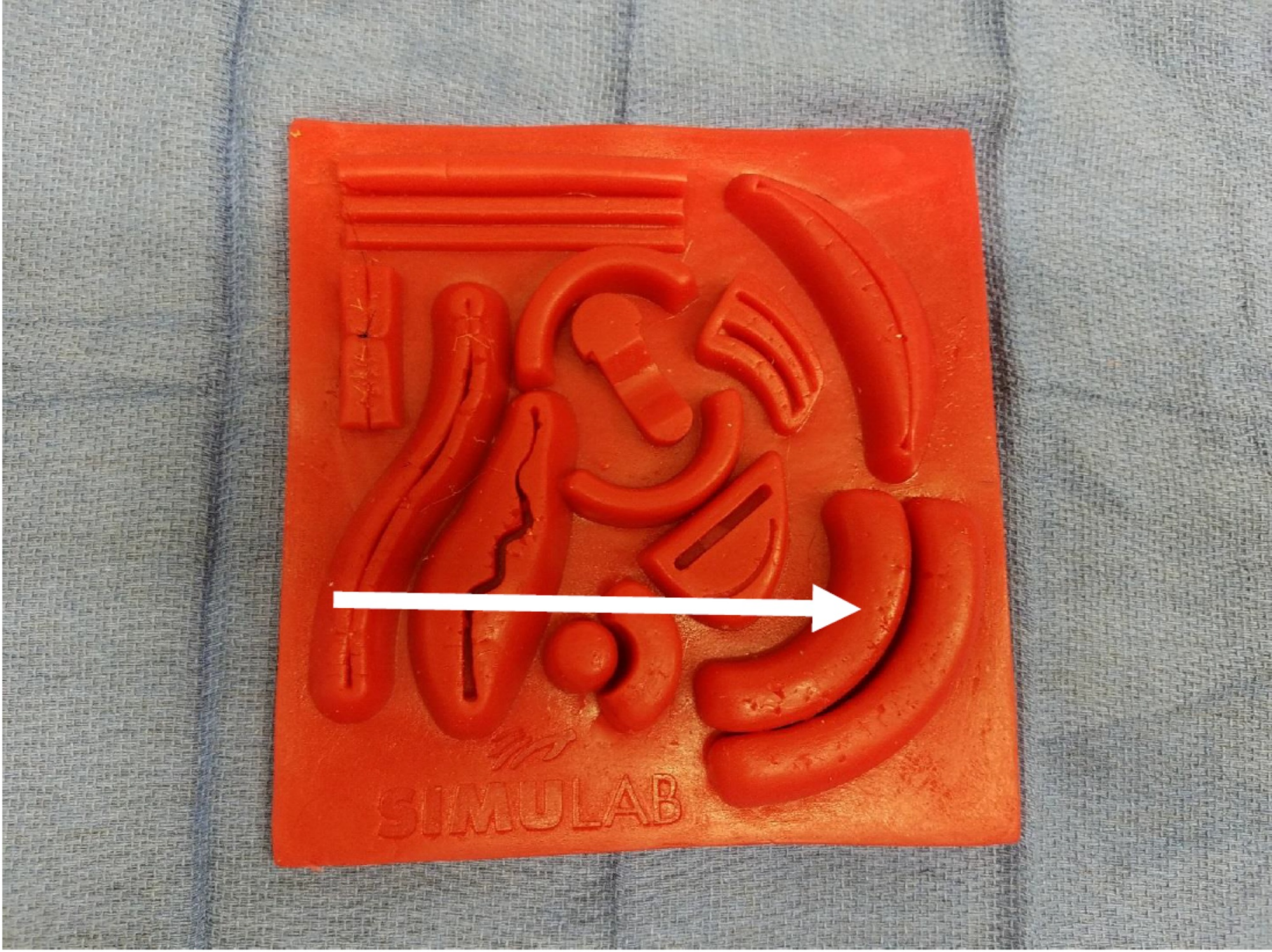
Instructed location of simulated laceration repair (indicated by arrow).

Subjects were given a case vignette of a child who required three simple, interrupted sutures to repair a laceration. They were presented with standardized equipment to perform the laceration repair, including two suture materials: fast absorbing plain gut and Vicryl (polyglactin 910) (Figure 3). Laceration repairs were performed on Tissue Suture Pads (SKU: TSP-10, Simulab Corp, Seattle WA) in a standardized location (Figure 4). Subjects were instructed to articulate individual steps of the procedure they were performing as they completed the laceration repair.

Videos of the laceration repair procedure were simultaneously recorded on two different cameras: one filming at a wide angle, the second filming with a zoomed-in view. The two videos were edited into one video comprised of elements of both recordings using video editing software (Movavi Video Editor 11, Movavi, St. Louis, MO, USA). The videos were filmed by authors NU and AT, and were edited by the study principal investigator (PI) (NU) to optimize views of the procedure. No components of the procedure were edited out of the videos.

Video scoring and data analysis

Eight physicians – five PEM attendings, two PEM fellows, and one emergency medicine attending – were recruited to evaluate and score study videos. Reviewers were familiarized with the rating tool, but did not receive training in its use. Five physicians (AT, RB, MJ, RK, JR) were from the study institution, while three (IG, RW, JU) were from outside centers. The study PI (NU) did not participate in video reviews. Video order was randomized, and then videos were randomly distributed to the reviewers. Reviewers then evaluated each video using the GRS tool. Each video was reviewed by at least five reviewers. The order of the videos was then randomized again, and each reviewer was sent videos to review using the checklist tool, with each video being once again reviewed by at least five reviewers.

For each study subject, overall GRS and checklist scores were averaged across reviewers. Average scores were compared by study subject level of training and previous self-reported procedural experience using linear regression. To assess agreement in scores across reviewers, concordance correlation coefficients (CCCs) were calculated for both the overall GRS and the overall checklist scores using variance components estimated from fitting linear mixed effects models [17,18]. CCCs were also calculated for each item for the GRS to evaluate agreement across reviewers for individual GRS items. Intraclass correlation coefficients (ICCs) were calculated using the variance components of generalized mixed effects models with a binomial distribution and logistic link function to determine agreement on individual checklist items [19-21]. Spearman's correlation coefficients were calculated to measure the association between the mean GRS and mean checklist scores for each proceduralist. For a sensitivity analysis, a linear mixed effects model was fit on 124 cases where the rater used both the GRS and the checklist for an individual study subject.

For the GRS, scores were tiered based on the mean performance per task, presuming a score on the last item, "Post-procedure" of five, as this item can only be scored a one or five. This resulted in following tiers: high performance (55–60, average score >4.5 on each item), good performance (49–54, average score >4 on each item), fair (39–48, average score >3 on each item), and remedial (12–38). Study subjects with individual reviewer scores in the lowest tier had their lowest and highest individual reviewer scores excluded and had their remaining mean scores compared to the mean GRS scores of all subjects with their lowest and highest scores excluded using a two-sided t-test. Statistical analyses were performed using SPSS Version 19 (IBM Co, Armonk, NY) and R Version 3.4.0 (R Foundation for Statistical Computing, Vienna, Austria). Study sample size was generated to detect a 10-point difference between first year residents vs second and third year residents in mean GRS scores with $\alpha$ = 0/05 and 80% power, presuming a mean score standard deviation of 9. This required the enrollment of 14 subjects in each group. Residents were split into two groups based on natural divisions in experience and to maximize study power. We *a priori *chose five video raters and 30 study subjects such that with the above standard deviation and 80% power we could detect an intraclass correlation coefficient of up to 0.89 between raters with 95% confidence, although CCC was ultimately used to assess agreement in scores across reviewers.

## Results

A total of 30 subjects participated in the study. Study subjects included 14 first year residents (interns), along with four second year residents and 12 third year residents (seniors). Twenty-eight participants self-reported previous laceration repair experience, while this data was not collected for two participants. Median intern experience was four procedures (Interquartile range (IQR) 4–12), median second year resident experience was 17.5 procedures (IQR 13.2–21.2), and median third year resident experience was 15 procedures (IQR 11.5–17.5).

**Figure 5:**
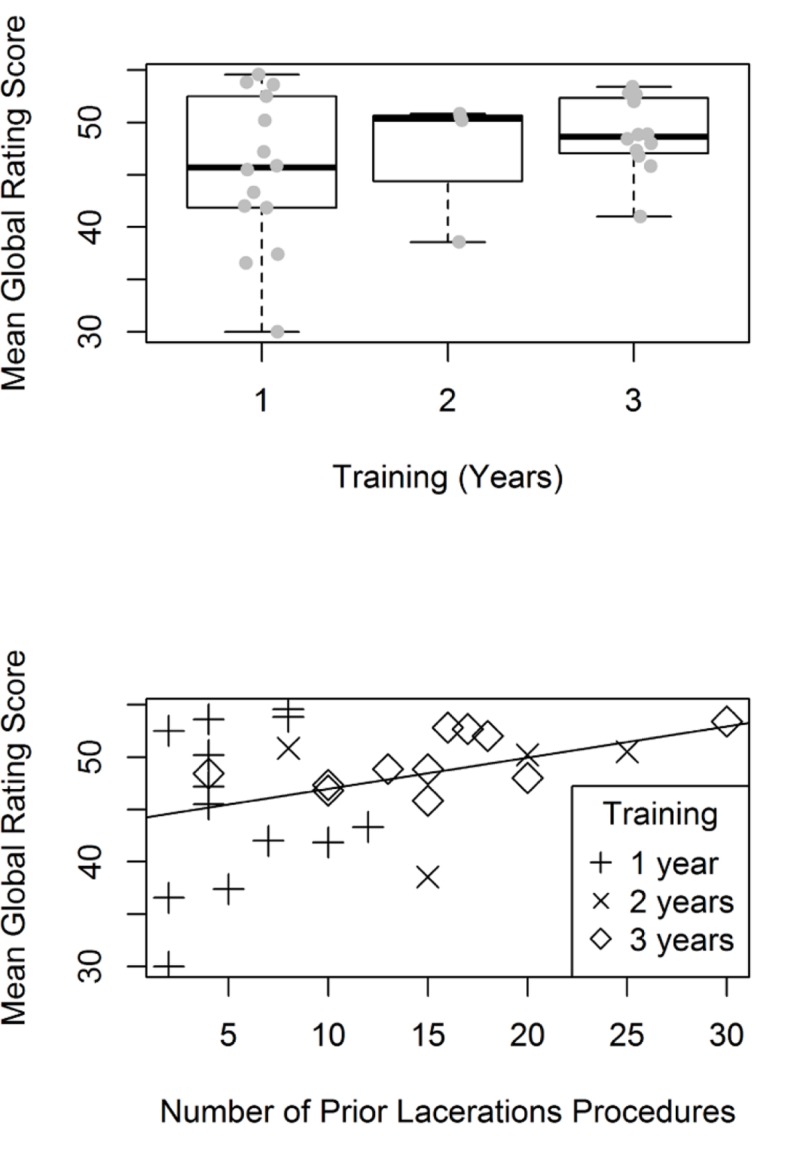
Mean global rating scale score by a) level of training and b) self-reported laceration repair experience.

**Figure 6:**
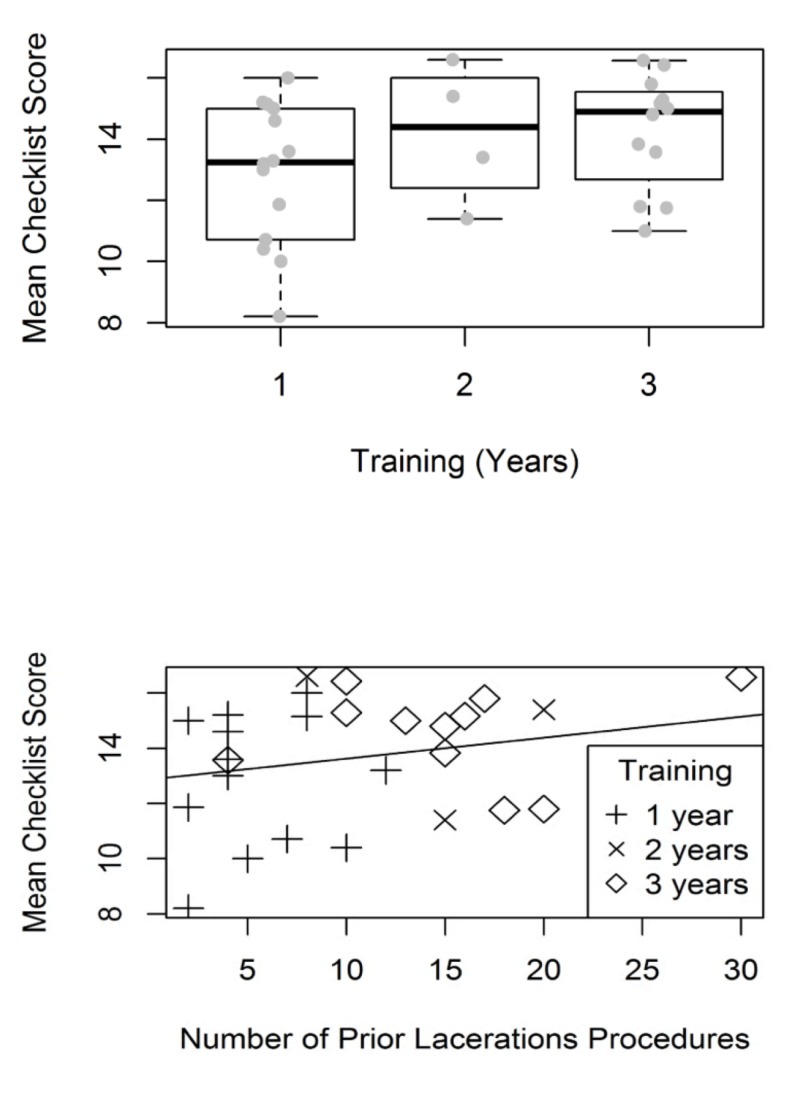
Mean checklist score by a) level of training and b) self-reported laceration repair experience.

There was no statistically significant difference in either GRS or checklist scores by years of training or procedural experience (Tables 1, 2). Figure 5 and Figure 6 show box plots of mean GRS and checklist scores by years of training and reported procedural experience. However, there were greater ranges in scores for the GRS and checklist tool for the more junior and less experienced trainees than in the more senior and more experienced trainees. Calculated CCCs showed fair concordance across reviewers for both the checklist (0.55, 95% CI: 0.38–0.69) and the GRS (0.53, 95% CI: 0.36–0.67).

**Table 1:** Mean proceduralist score by level of training.

	First Year Residents (n = 15), mean (SD)	Second and Third Year Residents (n = 15), mean (SD)	p-value
Global Rating Scale Score (max = 60)	45.3 (7.2)	48.5 (4.0)	0.15
Checklist Scale Score (max = 18)	12.8 (2.3)	14.2 (1.9)	0.09

**Table 2:** Expected increase in mean proceduralist score for every 10 procedures previously performed.

	Estimate	95% CI	p-value
Global Rating Scale (max score = 60)	3.0	(-0.1, 6.1)	0.06
Checklist Scale (max score = 18)	0.8	(-0.4, 1.9)	0.20

For each item on the GRS, mean scores for interns and seniors and the CCC for the item were calculated (Table 3). For each item on the checklist tool, mean percentages of interns and seniors completing each task and intraclass correlation coefficients were calculated (Table 4).

**Table 3:** Global rating scale item performance – for each item, mean scores were calculated for each proceduralist, then averages for each group. CCC: Concordance correlation coefficient

	Mean Score, First Year Residents	Mean Score, Second and Third Year Residents	CCC (95% CI)
Pre-procedure Preparation	3.84	4.19	0.35 (0.17-0.50)
Wound Anesthesia	3.76	3.76	0.60 (0.42-0.73)
Wound Irrigation	3.71	4.32	0.60 (0.42-0.73)
Use of Instruments	3.44	3.90	0.36 (0.18-0.51)
Time and Motion	3.46	3.59	0.30 (0.13-0.46)
Needle Insertion and Bite Size	3.44	3.85	0.32 (0.16-0.46)
Knot Tying	3.71	3.93	0.30 (0.13-0.45)
Self-correction	3.85	4.11	0.24 (0.08-0.38)
Overall Knowledge of Procedure	3.84	4.20	0.37 (0.20-0.52)
Independence	3.95	4.26	0.38 (0.21-0.52)
Overall Performance	3.61	3.98	0.40 (0.22-0.56)
Post-procedure*	4.78	4.45	0.12 (0.03-0.22)

**Table 4:** Checklist item performance – for each item, mean scores were calculated for each proceduralist, then averages for each group. ICC: Intraclass correlation coefficient

Item Number	Achieving Task, First Year Residents (% checked)	Achieving Task, Second and Third Year Residents (% checked)	ICC
Prepares for Laceration - 1	64%	71%	0.98
Positioning - 2	67%	65%	0.37
Anesthetizes - 3	56%	58%	0.20
Cleanses - 4	59%	83%	0.55
Maintains Sterility - 5	56%	68%	0.54
Use of Suturing Equipment - 6a	48%	56%	0.28
6b	85%	86%	0.53
6c	81%	82%	0.16
Needle Insertion - 7a	77%	77%	0.37
7b	72%	73%	0.23
7c	59%	66%	0.55
7d	55%	53%	0.06
Knot Tying - 8a	77%	86%	0.42
8b	82%	85%	0.93
8c	59%	56%	0.19
8d	60%	56%	0.28
Makes Corrections - 9	71%	80%	0.05
Discards Sharps - 10	83%	77%	0.13

There was a significant positive correlation in the mean GRS and mean checklist scores for each subject (Spearman ρ = 0.730, p < .01). In the linear mixed effects model for the sensitivity analysis, the association between the GRS and checklist scores was significant (p < 0.001).

For the GRS, scores were placed into descriptive tiers based on average scores per item (Table 5). Ten subjects had a score from at least one reviewer in the lowest tier (<39). Excluding their lowest and highest scores, these subjects had mean scores that were significantly lower than the overall mean score of all subjects excluding their lowest and highest scores (40.4 vs 47.3, p = .006).

**Table 5:** Suggested tiering of individual global rating scale scores.

Cutoff	Range	Tier	Mean Question Score at Cutoff	Comments
55	55+	High Performance	4.58	High performance on most items
49	49-54	Good performance	4.08	High performance on some items
39	39-48	Fair	3.25	Understands the basics, but needs oversight on most items
38 or less	12-38	Remedial		Needs oversight/instruction on most items

## Discussion

In this pilot study of a modified OSATS adapted to evaluate residents' suturing skills in a pediatric ED, we found the tool only had a fair concordance in scores across reviewers. The scores across the two components of the OSATS, the GRS and checklist, on average had a significant positive correlation, but there was inconsistency among reviewers. While scores increased by experience and training level, these increases were not significant. Based on this limited evidence of validity in internal structure and relationship to other variables the modified OSATS is not appropriate for summative testing in PED trainees.

The results of this study differ from a number of studies providing validity evidence for the use of the OSATS tool and its derivatives. The OSATS was initially developed for simulated assessment of surgical residents' technical skills. It showed good interrater reliability on both bench and live animal models for both the GRS and checklist scores (ranging from 0.64 to 0.72), providing evidence of its validity in these settings [4]. The OSATS was then adapted for use in multiple surgical and gynecologic settings. A review of surgical assessments of technical skills in 2010 found 26 studies that utilized some form of the OSATS tool [22]. Many of these studies showed construct validity, high internal consistency, and high inter-rater reliability. However, as validity of these constructs had not yet been translated to the operating room, the review's authors recommended using the tools only for formative feedback. Another review focused solely on the OSATS identified 29 studies utilizing this tool. They again found sufficient evidence to endorse use of the OSATS for formative feedback. However, an absence of evidence linking OSATS scores to performance in real clinical settings or regarding implications and decisions based on OSATS scores made the OSATS inappropriate for higher stakes decisions [23].

In pediatrics, the procedure for which the greatest development of procedural assessment tools has occurred is lumbar puncture (LP). Gerard et al. developed a tool consisting of a four-point GRS and a 15-item checklist to evaluate infant LP procedural performance with fair to good correlation in scores between raters [10]. Iyer et al. developed a seven-point GRS tool with similar interrater reliabilities [11].

The specific task of laceration repair had been previously evaluated by Acton et al. in medical students on a surgical rotation [7]. This study also used an OSATS-based tool with GRS and checklist components. This tool was validated by having 11 raters evaluate a demonstration examination, and checklist items with less than 75% agreement were revised. The authors found greater than 80% agreement for each of their seven GRS items. However, raters were considered in agreement if they were within ± one point of each other on a five-point GRS scale. Additionally the focus of the study was not validating the OSATS, but instead comparing trainee performance before and after the implementation of a new surgical curriculum.

In developing and attempting to validate our modified OSATS tool, we attempted to adhere to the model of construct validity as developed by Messick and applied to medical assessment by Downing [12,13]. The content of the evaluation tool was based on recognized resources for laceration repair education [12-14], and was reviewed by experts in PEM who offered their feedback on the tool's validity. It was designed to encompass the totality of the laceration repair process, and not just specific aspects of it. We analyzed the reliability of scoring by employing multiple reviewers and comparing their scores. We examined the internal consistency of scoring by comparing the independent scores of the GRS and checklists tools. We compared trainees' scores to training and experience, presumed proxies of technical proficiency. Our inability to establish evidence of validity in many of these examined domains makes our tool inappropriate for use in summative evaluation.

There are many potential reasons why the results we obtained examining our modified OSATS tool differed from the findings of other researchers. As the goal of our study was to develop a tool to discriminate between resident trainees in a PED, we limited our study enrollees to this population. Other studies have used enrollees with a broader range of experience levels [10]. While we did not find significant differences among groups based on level of training or reported clinical experience, these may be poor proxies for procedural skill [3].

Notably, while there was no overall difference in performance between interns and more senior trainees, the range of average scores on both the GRS and checklist tools was much greater for interns than more senior residents. It may be that individuals entering residency have a wide range of procedural competency which narrows as residents engage in specialty training. No proceduralists reported performing less than two laceration repair procedures. It may be in our population this may be sufficient experience for many learners to gain competence. Alternatively, the infrequency of laceration repair procedures in pediatric training, as opposed to in surgical practice, may lead to a reduced differential in skill in pediatric trainees, as opposed to surgical trainees. This lack of difference between study enrollees may also have resulted in lower CCCs, as the lack of true difference in the skill level of study enrollees resulted in more arbitrary scoring by raters.

We developed our modified OSATS tool with the hope it would be widely applicable. We therefore attempted to make the tool as standalone as possible. Reviewers did not receive additional training in the use of the tool. This potentially resulted in some of the observed variation in scores, as interpretation of the scoring tool was left to each reviewer. This may have been exacerbated by the subjective nature of many of the checklist GRS items, as well as variation in displayed technical skills by trainees within a simulated procedure when placing different sutures. Additionally, reviewers, trained in PEM and emergency medicine, may have less uniform practices and standards than surgical specialists. This may have led to more variation in scoring than seen in surgical OSATS tools. Orientation of reviewers to the standards for laceration repair and calibrating scoring standards may reduce the observed variability in scoring.

There also existed additional limitations specific to this study. While we attempted to create as high fidelity simulation environment as possible, differences between the task trainer and an actual patient care scenario may have limited the fidelity of our construct and therefore our ability to measure actual proficiency in laceration repair. Specific aspects of laceration repair, such as patient movement, ambient noise, lighting issue, and the stress associated with performing a procedure on an actual patient, could not be reproduced in our scenario. Additionally, reviewers scored filmed procedures, allowing multiple reviewers to review the same procedure. Review accuracy, however, may have been limited by video quality. We attempted to mitigate this concern by filming procedures from multiple angles and having trainees place three sutures. Nevertheless, video quality may have led to ambiguity in scoring. We attempted to blind video reviewers to the identity of filmed subjects. We did not film subjects' faces and attempted to remove identifiers from subjects. However, blinding may not have been one-hundred percent complete as reviewers may have recognized participants' voices. We utilized video reviewers external to the study institution to mitigate this concern, and there were no apparent differences in scoring between internal and external video reviewers. Nevertheless, undetected biases in scoring may have been present.

Finally, not every reviewer reviewed each video using each tool. This was done to both decrease the video reviewing burden for each reviewer and to minimize video repetition to allow for independent scoring. There may have been biases in scoring based on the particular reviewer assigned to each video. We attempted to mitigate this by randomizing video distribution and developing distinct randomization schemes for the GRS and checklist review procedures. This also somewhat limited our ability to perform exact comparisons between GRS and checklist scores. Despite this limitation, we were able to establish correlation between the scores obtained using these two tools.

While scores from the modified OSATS were not precise enough for summative evaluations, the tool may have utility for formative evaluation. While only fair, there was correlation between reviewers' scores. Averages scores between the GRS and the checklist tools also correlated. While not significant, there was a trend to higher scores in more experienced trainees, and specifically an absence of extremely low scores in more advanced trainees. Finally, proceduralists with individual GRS scores in the lowest scoring tier, determined a priori, had scores that were significantly lower than the GRS scores of all participants, excluding the highest and lowest scores received by each proceduralist in both groups. This means that receipt of GRS score in the lowest tier (<38) from an individual reviewer could potentially be used to identify proceduralists in need of additional attention to their training.

## Conclusions

We developed a modified OSATS tool for the evaluation of resident laceration repair performance in the PED and evaluated its validity. We based the tool on established resources for laceration repair and had experts in PEM review the tool to establish content validity. The tool showed fair concordance in scores across reviewers, but there was a significant correlation of scores between the GRS and checklist portion of the tool, providing some evidence of validity in internal structure. There were no significant differences in scores by self-reported laceration repair experience or level of training, limiting our ability to establish validity in relationship to other variables.

In this study the modified OSATS’ performance was inferior to its reported performance in the evaluation of surgical trainees, and it is therefore not appropriate for use in summative evaluation in the PED. The tool may have utility for formative feedback in this setting. Further study is needed to determine if question refinement and improved reviewer training would lead to greater score concordance.
